# Joint models in big data: simulation-based guidelines for required data quality in longitudinal electronic health records

**DOI:** 10.1186/s13040-025-00450-z

**Published:** 2025-05-13

**Authors:** Berit Hunsdieck, Christian Bender, Katja Ickstadt, Johanna Mielke

**Affiliations:** 1 Computational Biology, Bayer AG, Wuppertal, Germany; 2 Department of Statistics, TU Dortmund University, Dortmund, Germany; 3 Lamarr-Institute for Machine Learning and Artificial Intelligence, Dortmund, Germany

**Keywords:** Joint modelling, Longitudinal data application, Primary care data, Simulation study, Chronic kidney disease

## Abstract

**Background:**

Over the past decade an increase in usage of electronic health data (EHR) by office-based physicians and hospitals has been reported. However, these data types come with challenge regarding completeness and data quality and it is, especially for more complex models, unclear how these characteristics influence the performance.

**Methods:**

In this paper, we focus on joint models which combines longitudinal modelling with survival modelling to incorporate all available information. The aim of this paper is to establish simulation-based guidelines for the necessary quality of longitudinal EHR data so that joint models perform better than cox models. We conducted an extensive simulation study by systematically and transparently varying different characteristics of data quality, e.g., measurement frequency, noise, and heterogeneity between patients. We apply the joint models and evaluate their performance relative to traditional Cox survival modelling techniques.

**Results:**

Key findings suggest that biomarker changes before disease onset must be consistent within similar patient groups. With increasing noise and a higher measurement density, the joint model surpasses the traditional Cox regression model in terms of model performance. We illustrate the usefulness and limitations of the guidelines with two real-world examples, namely the influence of serum bilirubin on primary biliary liver cirrhosis and the influence of the estimated glomerular filtration rate on chronic kidney disease.

## Background

Identifying patients at high risk for clinical diagnosis as early as possible is increasingly important [1, 2]. Statistical models can be used to generate this early risk prediction of various health conditions. In this paper, we focus on the task of identifying patients at high risk for disease based on (longitudinal) biomarker levels. This is based on the hypothesis that small changes in biomarker levels can indicate changes in health, which ultimately lead to the diagnosis of a disease at a later time point [3].

Joint models, which combine longitudinal and survival data in a unified framework, present a compelling approach to take advantage of all available longitudinal information within a single model [4]. Research has shown that these models can enhance our understanding and yield improved parameter estimates compared to static survival data [5]. To implement joint models, it is essential to have a dataset that encompasses both types of data (survival and longitudinal data) of reasonably high quality.

Electronic health record (EHR) data, which typically includes longitudinal primary care and hospital information, offer a rich repository of patient health details, including laboratory results, diagnostic tests, treatments, symptoms and results [6]. However, working with EHR data presents numerous challenges, particularly in the pre-processing phase and in the analysis of processed data. The primary quality issues associated with the EHR data include incompleteness, inconsistency, and inaccuracy [7]. In the context of primary care data, specific challenges arise, such as missing data, noise, irregular data patterns, and the difficulty in accurately identifying relevant data points.

So far, it has not been systematically assessed how joint models perform in noisy real-world data as expected in EHR data sets, that is, what level of data quality is required so that joint models still offer an advantage in terms of precision and bias compared to other more established approaches such as Cox regression [8].

The primary objective of this paper is to establish guidelines for the necessary quality of longitudinal data for joint models through simulations. More concretely, this paper conducts an extensive simulation study systematically and transparently by varying different characteristics of longitudinal data, including measurement frequency, noise, and heterogeneity between patients. Utilizing the simulated data, we apply the joint models and evaluate their performance compared to traditional Cox survival modelling techniques. Insights gained in this simulation study are summarised in guidelines for data quality that other researchers can apply when making the decision whether a joint model or another model should be fitted.

We illustrate the usefulness of the guidelines with two practical examples to evaluate whether long-term biomarker records are of sufficient quality to extract insights from these trajectories. In particular, we examine how Bilirubin impacts primary biliary cirrhosis and how the estimated glomerular filtration rate (eGFR) affects chronic kidney disease (CKD).

## Methods

### Framework for simulating longitudinal primary care data

We present a simulation framework to generate realistic primary care and hospital data for joint modelling that will be used to investigate the impact of various characteristics of data quality on disease progression prediction models.

**Fig. 1:**
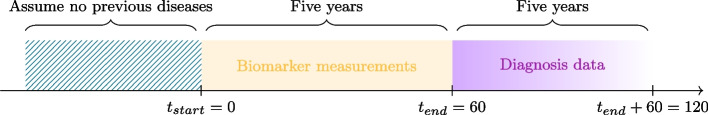
Scheme: Designated observation periods given in months for simulated data

For our simulation study, we assume that patients enter the study at time $t_{start}$ without prior diagnosis of the relevant disease. The patients are then followed over a 5-year observation period in which both survival data and longitudinal data are collected, ending at the time point $t_{end}$. Subsequently, starting after $t_{end}$, a 5-year follow-up period is considered, where no longitudinal data is observed and only survival data are recorded (see Fig. 1). Time is measured in months. We assume that the longitudinal data (the EHR or biomarker data) are scaled to lie within an interval of $\left[ 0,1\right]$, which can be achieved by min-max normalisation. We also assume a balanced design with a 50-50 split between healthy and diseased patients.

We generate the following types of data in our simulation:

1. Survival outcome, i.e., information if a patient is diagnosed or not during follow-up period
2. Longitudinal EHR data, e.g. measurements of biomarkers (from $t_{start}$ to $t_{end}$)
3. Fixed baseline characteristics of patients, such as sex and age (see Appendix 1, Simulation of the baseline characteristics section), that affect survival outcomes and are chosen differently between cases and controls so that they indirectly influence the survival outcomes.

In the following, we define $P_i$ as a patient *i*, $i=1,\ldots ,N$. For simplicity, we consider univariate longitudinal data, i.e., the availability of a single measured biomarker. Details of the data generation process are described in the following sections.

#### Survival outcome

The simulation of the diagnostic time point (time point of the event), denoted as $t_{i, abs}$, was carried out within a specified range of 10 to 119 months. This means that we require *some* longitudinal prior (i.e., between month 0 and month 10) to the event and that if a patient is to be diagnosed with the disease, this event would occur no later than five years after the biomarker observation period. For healthy patients, the diagnosis time point was right-censored at 120 months, indicating that no diagnosis was made within the observation period. In contrast, for patients who developed the disease, the diagnosis time point was randomly assigned based on a uniform distribution between 10 and 119 months,

1 $$\begin{aligned} t_{i, abs}\sim \left\{ \begin{array}{ll} 120 & \text {, if patient} i\ \text {is healthy patient (censored after 120 months)}\\ U(10,120) & \text {, if patient}\ i\ \text {becomes sick}, \end{array}\right. \end{aligned}$$

where *U* represents the uniform distribution. This shows that there is no specific increase or decrease in risk during the time window in which the GP data are modelled.

#### Longitudinal data

With the increasing availability of biobanks and EHR data, these resources can be utilised to enhance the development of predictive models. Especially longitudinal EHR data come with irregularities, such as the varying number of measurements and the differing precision of these measurements. To understand the impact of these distinct characteristics on predictive models, it is essential to simulate such data in a realistic and verifiable manner.

In EHR data, biomarker measurements are typically not taken at prespecified time points, but varying between patients (that is, taken on the decision of the physician). That is why, for the longitudinal data, we need to both simulate the data frequency and the actual data time and the values.

##### Number of Measurements

The number of measurements for each patient, denoted as $n_i$, is simulated using the absolute value of a normal distribution realisation with the mean number of measurements $n_{abs}$ per year and the standard deviation 2. This distribution is chosen to reflect the variability in the frequency of measurements observed in real-world EHR data and can help to analyse the influence of the number of measurement frequency on prediction. Figure 10 (in the Appendix 1) shows the mean number of measurements and the standard deviation for 66 biomarkers in the UK Biobank data (see the Appendix 1, UK Biobank section), focussing on data up to five years before the baseline visit. Based on this, a mean standard deviation of 2 and 1 measurement every two years on average is most likely for a typical biomarker. As we are interested in examining longitudinal data, the UK Biobank dataset can be tailored to include only those patients with a minimum of two to three measurements. This filtering increases the average number of measurements per year, but leads to a reduced sample size and potential biases. The number of measurements $n_i$ is simulated so that

2 $$\begin{aligned} n_i \sim \lfloor \mathcal {N}(n_{abs}\cdot \frac{t_{i,abs}}{12},2) \rfloor \end{aligned}$$

The diagnostic time point $t_{i,abs}$ is included in the expectation of the number of measurements because the total count of measurements depends on the duration up to the diagnosis, that is, if a longer time until diagnosis is observed, more longitudinal data can be measured.

##### Simulation of the Distribution of Measurement Dates

The measurement time points for the patient *i*, denoted as $t_{i,j}$, are simulated as random integers $\{t_{i,1},\dots , t_{i,n_i}\}$ with

3 $$\begin{aligned} t_{i,j} \sim U(0, t_{i,abs}), \ \ j\in \{1,\dots , n_i\}, \end{aligned}$$

where *U* reflects the uniform distribution. The chosen parameter reflects that longitudinal markers are observed for 60 months (from $t_{start}$ to $t_{end}$). The measurement dates for each patient were generated independently, resulting in a set of random time points for the patient.

##### Simulation of Measurement Values

The final goal of this simulation is to generate longitudinal EHR data, denoted as $y(t_{ij})$, prior to the disease onset. For simplicity, we focus on continuous measurements here. We adopt a linear mixed-effects model to represent the underlying trend in longitudinal data prior to the onset of the disease. Linear mixed-effects models, as introduced by [9], are widely recognised for their efficacy in modelling longitudinal trajectories (e.g., see [10]).

More concretely, we assume

4 $$\begin{aligned} y_{ij}=y(t_{ij}) = b_{i}+m_i\cdot t_{ij} \cdot \mathbb {1}_{t_{ij}\le t_{i,abs}-12\cdot t_m}+\epsilon _{i,j} \end{aligned}$$

for patient *i*, where $b_i$ reflects a patient-specific intercept and $m_i$ reflects a patient-specific slope that is added to the data starting from a breakpoint $t_m$. The error term $\epsilon _{i,j}$ is time-point specific. This means that the expected value of the longitudinal data is assumed to be constant until the break point $t_m$, where the biomarker will start to change and already give an indication of the later diagnosis, and then will increase linearly thereafter.

The slope parameter $m_i$ is simulated in a two-step procedure: For patients who are in the process of developing the disease, the probability of showing an effect before the onset of the disease based on longitudinal data (responding) is modelled using a Bernoulli distribution with a probability of $p_{resp}$, that is,

$$\begin{aligned} p_i\sim \left\{ \begin{array}{ll} 0 & \text {, if patient}\ i\ \text {is healthy}\\ Bern(p_{resp})& \text {, if patient}\ i\ \text {is sick} \end{array}\right. \ \ \ . \end{aligned}$$

For healthy patients, we assume that the slope is 0 in all cases. Then, the slope $m_i$ is calculated based on

$$\begin{aligned} m_i=p_i \cdot m_i^*, \end{aligned}$$

with

$$\begin{aligned} m_i^*\sim \mathcal {N}(\mu _m,\sigma _m^2), \end{aligned}$$

where $\mu _m$ and $\sigma _m$ represent the mean value and standard deviation, respectively. The values for $\mu _m$ and $\sigma _m$ are discussed in Results section. This approach takes into account the expected heterogeneity between patients, as not all patients are expected to show an association between longitudinal and survival data.

For the intercept, we assume that patients developing the disease already exhibit different baseline levels and define the difference as $\Delta _b$ (w.l.o.g.: $\Delta _b\in \left[ 0,0.5\right]$). Therefore, we simulate the intercept $b_i$ with a normal distribution with mean 0.5 and standard deviation $\sigma _b^2$, i.e.,

5 $$\begin{aligned} b_i\sim \left\{ \begin{array}{ll} \mathcal {N}(0.5+\Delta _{b},\sigma ^2_{b}) & \text {, if patient}\ i\ \text {is sick}\\ \mathcal {N}(0.5,\sigma ^2_{b}) & \text {, if patient}\ i\ \text {is healthy}. \end{array}\right. \end{aligned}$$

As measurements are consistently affected by noise, additional time-independent noise is included, represented by

6 $$\begin{aligned} \epsilon _{i,t}\sim \mathcal {N}(0,\sigma ^2_\epsilon ) \ \ \ . \end{aligned}$$

#### Parameter choices and practical example

This simulation approach allows an individual generation of realistic longitudinal data that reflects the different trajectories of healthy and diseased patients prior to the disease, allowing for adjusting different data quality parameters.

Since we want to examine how individual data quality parameters influence the performance of models, we vary the corresponding parameters. The parameter choices given in Table 1 are selected to explore various aspects of patient response and measurement variability with respect to their influence on model performance. For example, the number of measurements is selected to align with their distribution within the UK Biobank (see Fig. 10 in the Appendix 1).

**Table 1:** Parameter selections for further simulation and investigation of the impact of data quality metrics on risk prediction (In bold: Reference values)

Parameter	Parameter Annotation	Parameter Choices
Sample Size	*N*	$\{50, 200,\textbf{ 500}, 5000\}$
Noise Standard Deviation	$\sigma _\epsilon$	$\{0.05,0.075, \mathbf { 0.15}, 0.3\}$
Percentage of Patients Responding	$p_{perc}$	$\{0, 0.2, 0.5, 0.8, \textbf{1}\}$
Years of Assumed Slope	$t_m$	$\{1,\textbf{3},5\}$
Number of Measurements per Year	$n_{abs}$	$\{1,\textbf{2},3\}$
Intercept Difference	$\Delta _b$	$0, \{\mathbf {0.1}, 0.2\}$
Intercept Standard Deviation	$\sigma _b$	$\{\mathbf {0.05}\}$
Slope Mean	$\mu _m$	$\{\mathbf {0.005}\}$
Slope Standard Deviation	$\sigma _m$	$\{0.001, \mathbf {0.005}, 0.01\}$

Since the range of the standard deviation depends on the range of the mean, only one parameter is varied for both the slope *m* and the intercept *b*

**Fig. 2:**
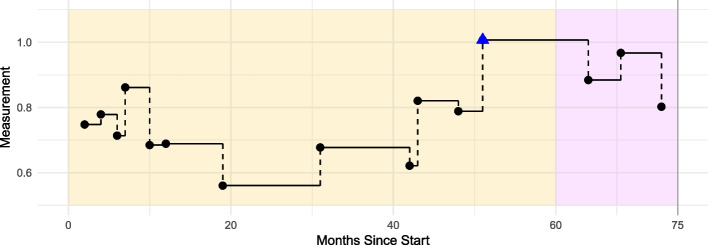
Example of a simulated trajectory of a sick patient: The Patient is getting sick after 75 months with twelve measurements during observation period $\left[ 0, 60 \right]$. The patient is male, not smoking and 66 years old. The blue triangle marks the last (simulated) measurements within the observation period

To keep the number of settings under evaluation in a manageable range, we use a reference setting per parameter (in bold in Table 1) and vary one parameter at a time. Parameter selections are designed to be based on Min-Max normalised values so that the values are within a $\left[ 0,1\right]$ range, facilitating transferability to real-world data settings. Since the range of the standard deviation depends on the range of the mean, only one parameter is varied for both the slope *m* and the intercept *b* (only one scenario for the other parameters). An example of a simulated patient trajectory for a patient diagnosed after 75 months is given in Fig. 2. It is evident that approximately five years before diagnosis, the values start to change exhibiting an increasing trend over time until the diagnostic time point at $t=75$. During the observation period $\left[ 0,60\right]$, a total of 12 measurements are available.

### Theoretical foundations of joint models and time-varying evaluation metrics

To systematically investigate the effects of different characteristics of quality and quantity of longitudinal EHR data on the predictive power of a joint modelling approach (see Joint model section), we compare the prediction performance with a standard Cox model. The approaches are evaluated by a version of the time-varying concordance index (see Evaluation of risk prediction models: adjusted time-varying concordance index). This allows for a comprehensive analysis of the influence of longitudinal data of varying quality on the prediction of the risk of disease progression. In the Cox model, the most recent known value is selected to overcome issues related to missing data. Conversely, the joint model is capable of addressing data gaps, as it models the entire trajectory based on the available information. This approach eliminates the need for imputation methods.

#### Joint model

Joint modelling of longitudinal and time-to-event processes enhances the precision of the estimation and predictive performance by effectively capturing the intrinsic relationships between the submodels. This approach is particularly advantageous in longitudinal studies, as it characterises the association between a longitudinal response process and a time-to-event outcome [4]. Consequently, these models have become increasingly popular in recent years and, as a widely utilised class of models, will serve as the foundation for developing the guidelines.

The joint model can be split into two submodels, the longitudinal model and the survival model. For the endogenous time-dependent longitudinal covariate (e.g., biomarker measurements), let $y_{ij}(t)$ be the observed value of the *i*-th subject at time point $t_{ij}$,

$$\begin{aligned} y_{ij}=\left\{ y_i (t_{ij}),j=1,\dots ,n_i\right\}. \end{aligned}$$

Let $T_i^\star$ be the true event time for the i-th subject and $T_i$ the observed event time with $T_i=min\left\{ C_i,T_i^\star \right\}$, $C_i$ the potential censoring time, and $\delta _i=\mathbb {1}(T_i^\star \le C_i)$ the event indicator.

##### Longitudinal Model

For the longitudinal model, we assume that the longitudinal outcomes are normally distributed and follow a linear shape. Then, the mixed-effects model is given by

$$\begin{aligned} y_i(t)&=m_i(t)+\epsilon _i(t)\\&=x_i^T(t)\beta +z_i^T(t)b_i +\epsilon _i(t) \end{aligned}$$

with

$$\begin{aligned} x_i(t), \beta&:\text {Fixed effects parts} \\ z_i(t), b_i&:\text {Random effects parts, } b_i\sim \mathcal {N}(0,D)\\ \epsilon _i(t)&: \text {Time-dependent error terms, } \epsilon _i(t)\sim \mathcal {N}(0,\sigma ^2) \end{aligned}$$

with variance-covariance matrix *D*. The longitudinal model is implemented using the R package nlme [11] that includes the estimation of the covariance matrix of the random effects.

##### Survival Model

For the survival model, the relative risk is given by

$$\begin{aligned} h_i(t|\mathcal {M}_i(t),w_i)= h_0(t)exp\left\{ \gamma ^Tw_i+\alpha m_i(t)\right\} ,\ \ t>0 \end{aligned}$$

with

$$\begin{aligned} \mathcal {M}_i(t) & :\left\{ m_i(s), 0\le s < t\right\} \\ & :\text {History of true unobserved longitudinal process up to time point t}\\ m_i(t) & : \text {True and unobserved value of covariate at time t (equivalent to respective part in longitudinal model)}\\ h_0(t) & :\text {Baseline risk function at time}\ t\\ w_i & : \text {(Vector of) baseline covariates with coefficients vector}\ \gamma \\ \gamma & :\text {Vector of regression coefficients for baseline covariates}\\ \alpha & :\text {Effect of underlying longitudinal outcome to the risk for an event}\\ & :\text {Quantifies association between time-varying covariate and risk of event} \end{aligned}$$

The survival model can be implemented using the R package survival [12].

##### Joint Distribution

Assuming that the two processes are associated, we can define a model for their joint distribution by assuming that we have full conditional independence, e.g., the random effects explain all interdependencies. This yields to the joint distribution

$$\begin{aligned} p(y_i,T_i,\delta _i)=\int p(y_i|b_i)\cdot \left\{ h(T_i|b_i)^{\delta _i} S(T_i|b_i) \right\} p(b_i) db_i \end{aligned}$$

with

$$\begin{aligned} b_i & :\text {Vector of random effects explaining interdependencies}\\ p(\cdot ) & : \text {Density function}\\ S(\cdot ) & :\text {Survival function} \end{aligned}$$

Taking into account the longitudinal submodel (see Longitudinal Model section) as well as the survival submodel (see Joint model section). The models are jointly optimised with the EM algorithm through Bayesian approaches using MCMC techniques [13]. The prior distributions are defined using frequentist univariate regression models fitted separately for each outcome. The mean of the Gaussian prior is defined as the maximum likelihood estimate (MLE) and the precision is defined as the inverse of 10 times the variance of the estimate from the univariate model. Regarding the prior distributions for the variance and covariance parameters of Gaussian random effects, JMbayes2 uses gamma priors with mean defined as the MLE of univariate models [14]. It is assumed that, based on the observed history, the mechanisms of censoring and the process of visiting are independent of the actual event times and future longitudinal measurements. This implies that the decision on the withdrawal of a subject from the study or their attendance at the clinic for a longitudinal evaluation is influenced by their past history, without additional causalities of the underlying latent characteristics of subjects that may be related to his prognosis.

In the following, the joint model will be consistently implemented and fitted using the R package *JMbayes2* [15].

#### Candidate models for comparison of performance

In this section, we compare the performance of the joint model in simulated data with different characteristics (as outlined in Framework for simulating longitudinal primary care data section) with the Cox model, as the standard approach for modelling survival data. We aim to identify settings in which the more complex joint model outperforms the Cox model. The models to be compared are given by

1. Joint model incorporating biomarker measurements from the past 5 years along with covariates such as sex, age, and smoking status (represented in green in the Results section). More concretely, the submodels are given by:
  $$\begin{aligned} y_i(t) & =m_i(t)+\epsilon _i(t)\\ & =\beta _0+\beta _1 t+ \beta _2 {Sex_i}+ \beta _3 {Age_i}+\beta _4 SmokingStatus_i+b_{i0}+b_{i1}t+\epsilon _i(t)\\ h_i(t) & =h_0(t)\cdot exp\{\gamma _1 {Sex_i}+ \gamma _2 {Age_i}+\gamma _3 SmokingStatus_i+\alpha m_i(t)\} \end{aligned}$$
  where the parameters $b_{i\cdot }$ mark the individual subject-specific effects.
2. Cox model including covariates such as age and sex, but no EHR data (“baseline model”, shown in blue in the Results section).
  $$\begin{aligned} h_i(t)=h_0(t)\cdot exp\{\gamma _1 {Sex_i}+ \gamma _2 {Age_i}+\gamma _3 SmokingStatus_i\} \end{aligned}$$
3. Cox model that incorporates the covariates of age and sex along with the most recent measurement $\tilde{y}_i$ of the biomarker in the 5-year observation period (represented in orange in the Results section).
  $$\begin{aligned} h_i(t)=h_0(t)\cdot exp\{\gamma _1 {Sex_i}+ \gamma _2 {Age_i}+\gamma _3 SmokingStatus_i+\gamma _4 \tilde{y}_i\} \end{aligned}$$

It is important to note that the Cox model uses biomarkers measured at $t_{end}$, while additional longitudinal data for the joint model is obtained prior to $t_{end}$. With this selection, we ensure a fair comparison between the models.

Table 1 lists the various scenarios that will be examined.

#### Evaluation of risk prediction models: adjusted time-varying concordance index

The goal is to evaluate the precision and reliability of risk models. More concretely, we aim to identify with highest accuracy those who are at increased risk of developing a disease. Commonly used scores to evaluate the performance of the model are the C index to evaluate the model’s ability to rank the risk of the subject and the integrated Brier score incorporating the discrimination and calibration aspect [16]. Forecasting risk is largely affected by the specific time period being examined (for instance, a Cox model can “*more readily*” evaluate risk profiles over a brief period like a month rather than spanning several years). For further evaluation, we introduce the time-varying C-index, derived from the conventional C-Index (see [17]). This metric can be readily understood in terms of time and adapted according to varying risks over different periods.

**Fig. 3:**
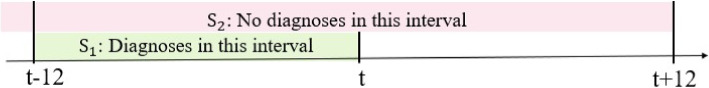
Illustration of subgroups $S_1$ and $S_2$ for the time-varying C-Index definition

Given the estimated individual risk $r_i(t)$ at time t of patient *i*, we define two subsets as illustrated in Fig. 3 by

$$\begin{aligned} S_1= \bigl \{\text {Individuals with diagnosis in interval } \bigl [t-interval,t\bigr ]\bigl \} \end{aligned}$$

and

$$\begin{aligned} S_2= \bigl \{ \text {Individuals without diagnosis in interval } \bigl [0,t+interval\bigr ]\bigl \} \end{aligned}$$

with $|S_1|=n_1$ and $|S_2|=n_2$. The time-varying C-index is then defined as

7 $$\begin{aligned} tvC_{interval}(t)=\frac{\sum _{k\in S_1} \sum _{l\in S_2}\mathbb {1}_{\{r_k(t)>r_l(t)\}}}{n_1\cdot n_2} \ \ \ \in \left[ 0,1\right] \end{aligned}$$

which expresses the ratio of pairs in which the predicted risk for a pre-disease patient (subset $S_1$) exceeds the predicted risk for a temporarily healthy patient (subset $S_2$), relative to all possible pairs. The closer the time-varying C-index is to 1, the better the model’s performance. Compared to the established C-index, this measure allows us to observe changes in the performance over time.

For a observation period of five years ($t_{end}=60$) and a follow-up period of five years ($\Delta t=60$), the mean time-varying C-index of the follow-up period $[t_{end},t_{end}+\Delta t]$ is given by

8 $$\begin{aligned} mean(tvC_{interval}(t))=\frac{1}{\Delta t}\sum _{t= t_{end}}^{t_{end}+\Delta t} tvC_{interval}(t)=\frac{1}{60}\sum _{t= 60}^{120}tvC_{interval}(t) \end{aligned}$$

with *t* given in months.

#### Set-up and evaluation of simulation study

Models are compared with the introduced performance measure across 100 iterations of simulated data with the parameter settings described in Table 1. From these, the mean and the 0.05 and 0.95 quantiles are computed to establish the prediction interval for one dataset. Note that in the Results section, the prediction intervals are only depicted when a change in the prediction interval is given by different parameter choices; otherwise, they are omitted for the sake of clarity.

In the following, we consider the mean time-varying C-index for patients who remain at risk by $t_{end}$ (those not diagnosed until $t_{end}$) in three different models during the 5-year follow-up period. Therefore, patients with an early diagnosis, which can technically not be included in the Cox model analysis, are excluded from the evaluation to ensure fairness while comparing the joint model and the Cox model (see Candidate models for comparison of performance section).

### Software versions

The following software has been used:

**R Version** [18]**:** 4.2.1

**Package Versions:** dplyr [19]: 1.1.4; ggplot2 [20]: 3.5.1; JMbayes2 [15]: 0.5-0; patchwork [21]: 1.2.0; purrr [22]: 1.0.2; tidyr [23]: 1.3.1; MatchIt [24]: 4.5.5

## Results

### Derivation of simulation-based guidelines for longitudinal primary care data

In the following section, we examine how individual data quality parameters influence the performance of the joint model compared to the standard Cox model [8], see Candidate models for comparison of performance section for details. For that, we discuss the impact of different parameter choices on the performance given by the 5-year mean time-varying C index (short: mean C-Index) after the last possible measurement time point $t_{end}=60$ with an interval length of 12 months. An interval of 12 months is selected to thoroughly examine the significant distinctions in risk.

Additionally, we evaluated two additional parameters, namely response rate and slope variance, over time (see Response rate and slope variance over time section) to demonstrate how performance differences evolve over time.

**Fig. 4:**
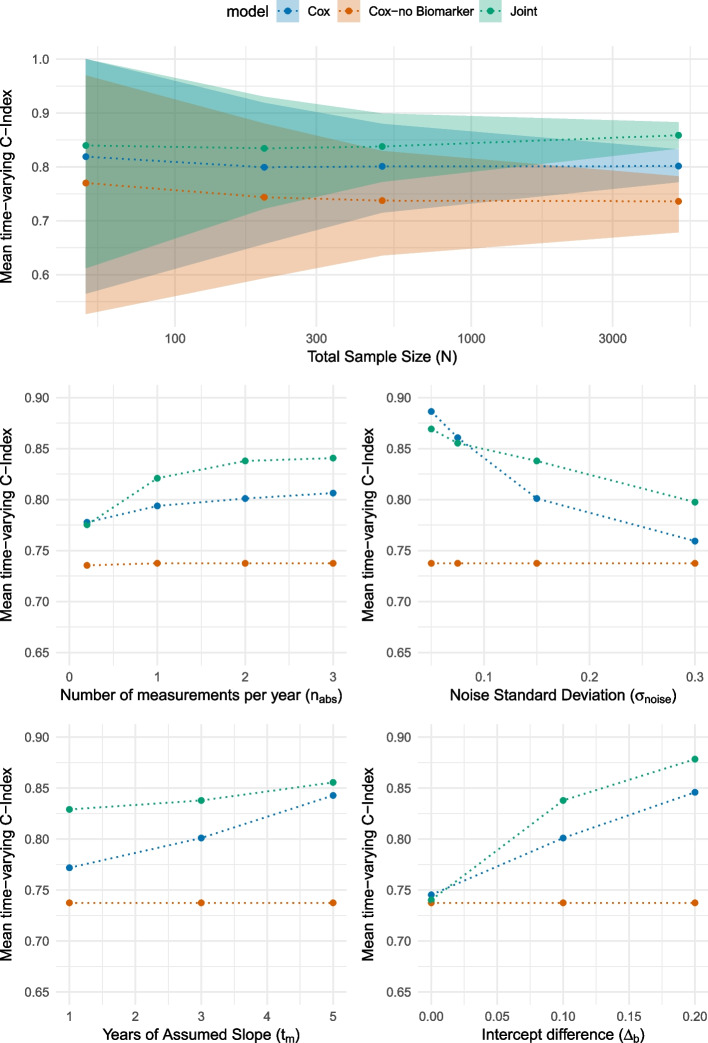
Comparison of the mean time-varying C-index over five years with an interval of 12 months for different parameter choices for the total sample size (**a**), the number of measurements per year (**b**), the noise standard deviation (**c**), the years of assumed slope (**d**), and the intercept difference (**e**)

Figure 4 illustrates the performance given by the mean C index of the three different models in the scenarios described in Table 1. We first note in general that the Cox model and the joint model outperform -as expected- the Cox model without biomarkers in all scenarios. Thus, we use the result from the Cox model without biomarkers for illustration of the baseline performance, but do not discuss it in detail and focus on the two other models. For clarity of the visualisations, we only provide prediction intervals for one scenario, but note that comparable variability is also present in the other cases.

#### Sample size

The available sample size in practice depends greatly on the specific use case. For example, without taking into account the availability of longitudinal biomarker data, the number of patients who are diagnosed with stroke (approx. 22.200 patients) is by far larger than the number of patients who are diagnosed with encephalitis (approx. 990 patients) within the UK Biobank. In our simulation study, we thus investigate the time-varying C-index for sample sizes between 50 and 5000 subjects. Figure 4a illustrates that in small sample sizes ($N=50$), the Cox model (orange) performs very similar to the joint model (green). However, the differences become more prominent when the sample size is increased to $N=200$. We also notice that there are further small gains in performance for the joint model compared to the Cox model - however, these differences are rather small. Therefore, we recommend a sample size of $N\ge 200$ for a robust prediction.

We also note that the prediction intervals for the mean C-index narrow with increasing sample size.

It must be considered that the recommended sample size is influenced by other factors and can therefore vary, for example, with respect to the homogeneity of the longitudinal data within the cohort under consideration.

#### Number of measurements (per year)

It is recognised that the number of measurements in the electronic health record (EHR) data can vary considerably depending on the specific marker and the effort required for measurement. Figure 4b illustrates the performance of the models with respect to different quantities of measurements. When there is at least one measurement per year, the joint model exhibits superior performance. However, after a certain threshold, additional data points do not provide further information and thus do not enhance performance. Consequently, it can be concluded that a greater number of measurements facilitates improved longitudinal trajectory modelling, thus enhancing risk prediction. We recommend at least 1 measurement per year if a joint model is to be used.

#### Varying noise variance

Next, the impact of the noise of the measurements itself is analysed. External factors, such as varying conditions that affect blood pressure readings, can significantly influence measurement noise, affecting model performance. In Fig. 4c, the importance of noise variance as a critical parameter is highlighted. It is observed that as the level of noise increases, the joint model demonstrates a greater advantage in the C-index over the Cox model. In contrast, at low noise levels (0.05), there is a minimal difference in performance between the Cox model and the joint model. The joint model is capable of filtering out higher levels of noise, yielding more accurate predictions in comparison to the Cox model. Starting at a noise variance of approx. $\sigma _e=0.075$, the joint model starts to perform better compared to the Cox model; therefore, we recommend the joint model specifically in those scenarios with at least $\sigma _e > 0.075$.

#### Years of slope

Typically small changes in biomarker level proceeds the clinical diagnosis. However, it is not necessarily clear * how* these changes are present much earlier. As shown in Fig. 4d, when the slope is given for a shorter time period (i.e., the biomarker starts to change only for a short period prior to the actual diagnosis), it is detected by the joint model but not by the Cox model. In contrast, when the slope is assumed to be over a longer duration (e.g., 5 years), there is only a minimal difference in the performance between the models. This can be explained by considering that the Cox model uses only the most recent measurement. If the slope begins to increase only recently prior to the most recent measurement used, the absolute difference in biomarker levels is smaller. Since the joint model is estimating the slope, even small differences can be retrieved. It is important to note that for practical application, this value is difficult to derive.

#### Intercept/baseline difference

Lastly, the impact of a baseline difference is analysed. Like covariates, a specific baseline difference in the (simulated) marker itself can occur within the risk cohort, independently of a specific time frame slope [25]. As illustrated in Fig. 4e, in scenarios without a difference in intercept, the Cox model and the joint model perform similarly. The joint model benefits more than the Cox model from an increase in the intercept. Therefore, the joint model appears specifically suitable if the intercept difference is greater than $\Delta _b \ge 0.1$.

#### Response rate and slope variance over time

**Fig. 5:**
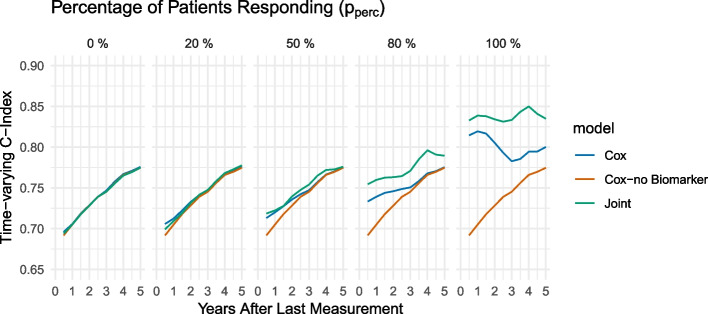
Comparison of the effect of different parameter choices for the fraction of patients responding $p_{resp}$ in simulated data on the time-varying C-index with an interval of 12 months using a joint model and a Cox model with/without biomarker information: $p_{resp}\in \{0, 0.2,0.5,0.8,1\}$

For specific parameters, not only the parameter itself but also time plays a role in its influence on performance, for example, the statistical model may be very good at predicting the two-year risk of diagnosis, but may fail to predict the five-year risk of diagnosis. Therefore, we look at the time-varying concordance index $tvC_{12}(t)$ over a period of time of 5 years after $t_{end}$ for varying choices of the fraction of patients with a relationship between EHR data and survival outcome ($p_{perc}$) and slope variance ($\sigma _m$). Figure 5 shows the performance of the different models based on the percentage of subjects who actually demonstrate the hypothesised relationship between the biomarker and the diagnosis. We note that only if at least a majority of patients (approximately 80%) demonstrate a response, the joint model or the Cox model (with biomarker) are superior to the baseline Cox model. Therefore, a highly heterogeneous relationship between the biomarker and the diagnosis makes it difficult to detect this pattern.

**Fig. 6:**
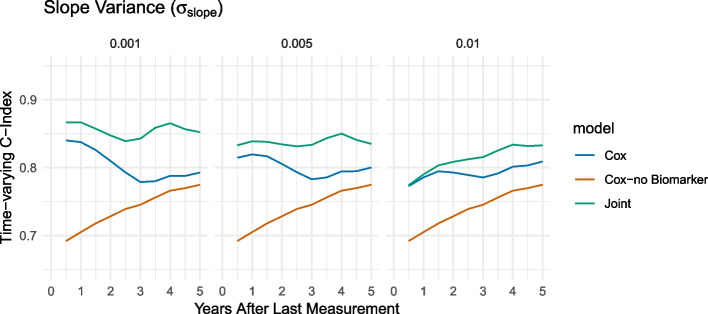
Comparison of the effect of different parameter choices for the slope standard deviation $\sigma _{m}$ in simulated data on the time-varying C-index with an interval of 12 months using a joint model and a Cox model with/without biomarker information: $\sigma _m\in \{0.001, 0.005, 0.01\}$

In Fig. 6, we compare different levels of variability of individual slopes. We note that the advantage of the Cox model is highest if the variability of the slope is small, i.e., the slope is similar across patients. However, as heterogeneity increases, the performance of the Cox model, including (bio)marker information, becomes more comparable.

When we consider the dependency of the performance on the chosen time points for the time-varying concordance index $tvC_{12}(t)$, we note that generally, for both parameters, the models are more similar for earlier time points (small values of *t*). This means that if the aim is to identify subjects at risk for a diagnosis at a time point that is close to the time of the last measurement, there is no strong advantage of the joint model compared to the Cox model. However, when comparisons are made at later time points, the joint model may begin to diverge from the Cox model because of its ability to predict and fit the longitudinal trajectory. This results in a more pronounced distinction between the joint and Cox curves, as illustrated in Figs. 5 and 6. The “bend” after approximately three years can be explained by the simulation of data that accounts for a biomarker change starting three years before the disease diagnosis, allowing the risk prediction to benefit from biomarker data within these three years.

#### Simulation-based guidelines for longitudinal primary care data

The well-established Cox model is much easier to apply and communicate than the more complex joint model. Therefore, we recommend the joint model only in scenarios in which the joint model outperforms the Cox model. Synthesising the findings of the previous sections, guidelines can be formulated that help to determine the utility of the available longitudinal Primary Care/EHR data for the identification of disease risk. The guidelines are presented in Table 2. To adhere to these guidelines, it is essential to normalise the longitudinal measurements to a range of [0, 1], ensuring a mean value of approximately 0.5, primarily for scaling purposes. Almost all of the listed parameters can be directly extracted from the available real-world data of interest. It is important to note that the response rate is typically unknown. To achieve a high response rate, the cohort can be restricted to specific subgroups of patients and/or disease. In the following section, two real-world data examples are analysed using the derived guidelines.

**Table 2:** Guidelines: criteria for normalised electronic health record data that preferentially support the joint model over the Cox model

Parameter	Superior performance of joint model compared to Cox model
Sample Size	$N\ge 200$
Noise Standard Deviation	$\sigma _\epsilon > 0.075$
Percentage of Patients Responding	$p_{perc}\ge 80\%$
Number of measurements per year	$n_{abs}\ge 1$
Intercept difference	$\Delta _b\ge 0.1$
Slope Standard Deviation	$\sigma _m\le 0.005$

### Case study: real-world applications of the derived guidelines

Table 2 presents guidelines formulated from simulated data. To demonstrate their applicability in real-world scenarios, two different datasets were subsequently examined using these guidelines.

#### Serum bilirubin and primary biliary cirrhosis (Mayo Clinic)

Primary biliary cirrhosis of the liver (PBC) is considered a progressive disease. The progression is slow and the resulting inflammation progressively results in cirrhosis, damage to the liver’s bile ducts, and ultimately death of the patient [26]. More information is available in Dickson et al. [27] and Markus et al. [28]. The data used for further modelling were collected from the Mayo Clinic trial on PBC that took place from 1974 to 1984. A total of 424 patients with PBC, referred to the Mayo Clinic during that 10-year interval, met the eligibility criteria for the randomised placebo controlled trial of the drug D-penicillamine. The first 312 cases in the data set participated in the randomised trial and contain largely complete data [12]. The data set for the randomised trial, consisting of 312 participants, is provided by the JMbayes2 R package [15].

The diseased subgroup comprises all patients who received an initial diagnosis of PBC within the 10-year interval. We used a 5-year period in which longitudinal data is collected, followed by a 5-year follow-up period in which only survival outcome data are recorded. Given these data, the guidelines of Table 2 are used to assess if better performance of the joint model compared to the Cox model can be expected in this scenario. Bilirubin values were initially converted using a logarithmic transformation, followed by a min-max transformation to normalize the distribution within a range of 0 to 1, targeting an average of approximately 0.5 for optimal application in guidelines. The normalised bilirubin value at time t is denoted as $bil_{norm}(t)$. The appendix outlines the process of deriving parameters using real-world data (see “Derivation of parameter choices based on real-world data” section in Appendix 1). For applying this approach to derive slope and noise parameters, it is assumed that $t_m = 3 \ \text {years}$ since PBC is progressing slowly. A summary of the derived parameters is given in Table 3.

**Table 3:** PBC dataset (See R package *jmbayes*2 for more information): estimation of parameters, derived by a mixed-effects model (see Appendix 1, Derivation of parameter choices based on real-world data section), and comparison with guidelines given by Table 2

Parameter	Estimate	Comparison to Guidelines
Sample Size *N*	312	Requirement fulfilled
Noise Standard Deviation $\sigma _\epsilon$	0.078	Requirement fulfilled
Percentage of Patients Responding $p_{perc}$	unclear	unclear
Number of measurements per year $n_{abs}$	1.6(*sick*), 1.4(*healthy*)	Requirement fulfilled
Intercept difference $\Delta _b$	0.116	Requirement fulfilled
Intercept standard deviation $\sigma _b$	$0.148 \ (0.124)$	-
Slope Mean $\mu _m$	0.007	-
Slope Standard Deviation $\sigma _m$	0.003	Requirement fulfilled
Years of *Assumed* Slope $t_m$	3	unclear

All requirements given in the guidelines are met. Therefore, we expect a gain in performance for the joint model compared to the Cox model.

For the joint model, the corresponding submodels are given by

9 $$\begin{aligned} & \text {Longitudinal submodel:}\nonumber \\ & \qquad \qquad \qquad bil_{norm}(t)=\beta _0+\beta _1\cdot t+\beta _2\cdot Sex+\beta _3\cdot Age+b_{i0}+b_{i1}\cdot t+\epsilon _i(t)\end{aligned}$$

10 $$\begin{aligned} & \text {Survival submodel:}\nonumber \\ & \qquad \qquad \qquad h_i(t)=h_0(t)\cdot exp\{\gamma _1 Sex_i+\gamma _2 Age_i+\alpha m_i(t)\} \end{aligned}$$

with fixed and random effects parameters of the longitudinal model similar to Longitudinal Model section. The Cox models are given by

11 $$\begin{aligned} \text {Cox model with biomarker} & \ \text {information:}\nonumber \\ h_{i,Cox_{biom}}(t) & =h_0(t)\cdot exp\{\gamma _1 Sex_i+\gamma _2 Age_i+bil_{norm}(\tilde{t})\}\end{aligned}$$

12 $$\begin{aligned} \text {Cox model without biomarker} & \ \text { information:}\nonumber \\ h_{i,Cox}(t) & =h_0(t)\cdot exp\{\gamma _1 Sex_i+\gamma _2 Age_i\} \end{aligned}$$

with $\tilde{t}$ denoting the last observation time point of a Bilirubin value within the observation period.

**Fig. 7:**
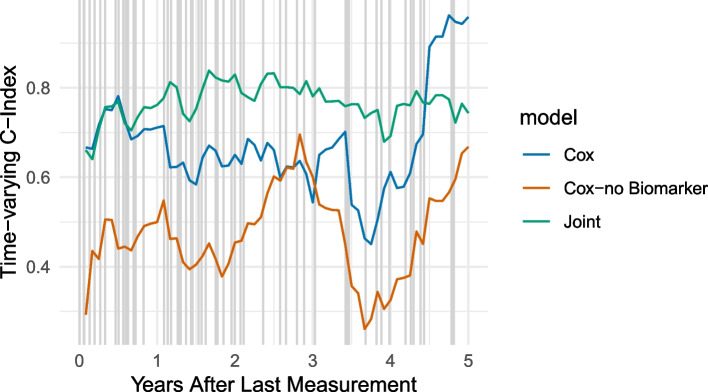
Assessment of Serum Bilirubin and Primary Biliary Cirrhosis (Mayo Clinic): Evaluation of different models (joint model, and Cox model with/without biomarker information) using the time-varying C-index over a period of five years with an interval of 12 months. Vertical grey lines mark diagnosis time points

As a result, we first note that the analysis reveals that the inclusion of biomarkers in the model provides a relevant information gain (Cox - no biomarker, illustrated in orange, substantially worse performance to the other models). When comparing the performance of the cox model with the joint model, it is confirmed, as illustrated in Fig. 7, that there is a gain in prediction accuracy for the joint model compared to the cox model if the prediction interval is longer than 1 year. This is in line with our expectations, since all criteria in the guidelines were met.

#### eGFR and chronic kidney disease (UK Biobank)

Data provided by the UK Biobank (UKB) (see Appendix 1, UK Biobank section) provide a valuable resource for research on chronic kidney disease (CKD). Estimated glomerular filtration rate (eGFR) measurements are well established to predict the risk of CKD, and eGFR is often used for diagnostic purposes in this context [29, 30]. It is of particular interest to investigate whether eGFR trajectories provide additional information about the diagnosis of CKD. To this end, UKB data will be used to implement a joint model and analyse data quality using the derived guidelines (see Table 2).

The initial step in the data preparation process involves general preprocessing, which includes joining units, converting measurements, and addressing implausible values and outliers (see the Appendix 1, Preprocessing of eGFR trajectories within UK Biobank GP data section). Chronic kidney disease (CKD) is diagnosed according to the ICD-10 codes detailed in the Appendix 2, as shown in Table 6, as specified by [31]. The included data are restricted to subjects with available covariate information and at least one measurement per year on average and a total of three measurements available during the 3-year period.

The diseased subgroup comprises all patients who received an initial diagnosis of chronic kidney disease (CKD) within three years prior to and up to five years after their visit to the assessment centre (visit V0 in UKBB). To create a comparable, similar-sized subgroup within the healthy cohort, propensity score matching is applied using the R package *MatchIt *[24], utilising Mahalanobis distance and the covariates age (UK Biobank Field ID: 21022), sex (UK Biobank Field ID: 31), and smoking status (UK Biobank Field ID: 20116). As a result, no covariates were incorporated into the longitudinal and survival submodel.

In this example, we use a 3-year period in which longitudinal data is collected, followed by a 5-year follow-up period in which only survival outcome data are recorded. The eGFR values are transformed using a Min-Max normalisation to obtain a distribution within a range of 0 to 1, targeting an average of approximately 0.5 for optimal application in guidelines. The normalised eGFR value at time t is denoted as $eGFR_{norm}(t)$.

Given these data, the guidelines of Table 2 are used to assess if better performance of the joint model compared to the Cox model can be expected in this scenario. For applying this approach to derive slope and noise parameters, it is assumed that $t_m = 1 \ \text {year}$. A summary of the derived parameters is given in Table 4. We note that some requirements are fulfilled (e.g. sample size) whereas other requirements are not fulfilled (e.g., noise is low, low number of measurements per year). Thus, it is unclear if a gain in performance is expected from the joint model.

**Table 4:** eGFR and chronic kidney disease: Estimation of parameters, derived by a mixed-effects model (see Appendix 1, Derivation of parameter choices based on real-world data section), and comparison with the guidelines given by Table 2

Parameter	Estimate	Comparison to Guidelines
Sample Size *N*	5272	Requirement fulfilled
Noise Standard Deviation $\sigma _\epsilon$	0.042	Requirement not fulfilled
Percentage of Patients Responding $p_{perc}$	unclear	unclear
Number of measurements per year $n_{abs}$	1.2(*sick*), 0.8(*healthy*)	Requirement not fulfilled
Intercept difference $\Delta _b$	0.119	Requirement fulfilled
Intercept standard deviation $\sigma _b$	0.082, 0.073	-
Slope Mean $\mu _m$	$-0.004$	-
Slope Standard Deviation $\sigma _m$	0.004	Requirement fulfilled
Years of Assumed Slope $t_m$	1	unclear

For the joint model, the corresponding submodels are given by

13 $$\begin{aligned} \text {Longitudinal submodel:} & \nonumber \\ eGFR_{norm}(t) & =\beta _0+\beta _1\cdot t+b_{i0}+b_{i1}\cdot t+\epsilon _i(t)\end{aligned}$$

14 $$\begin{aligned} \text {Survival submodel:} & \nonumber \\ h_i(t) & =h_0(t)\cdot exp\{\alpha m_i(t)\} \end{aligned}$$

The Cox model with biomarker information is given by

15 $$\begin{aligned} h_{i,Cox_{biom}}(t) = h_0(t)\cdot exp\{eGFR_{norm}(\tilde{t})\} \end{aligned}$$

with $\tilde{t}$ denoting the last observation time point of a eGFR value within the observation period. Since propensity score matching was performed, no covariates are included in the model.

**Fig. 8:**
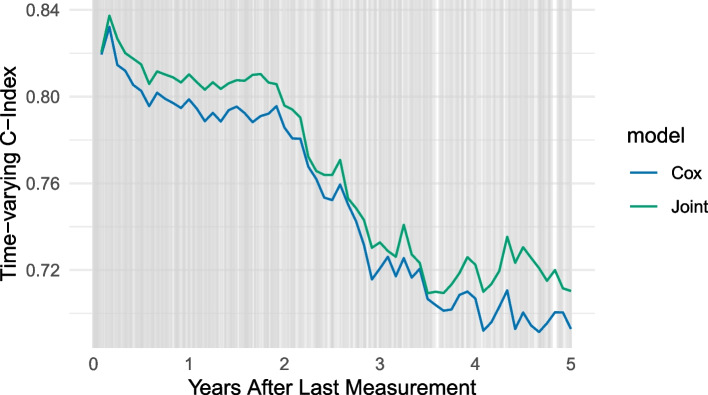
Assessment of eGFR and Chronic Kidney Disease in the UK Biobank: Evaluation of different models (joint model, and Cox model with biomarker information) using the time-varying C-index over a period of five years with an interval of 12 months. Vertical grey lines mark diagnosis time points

We note that there is no gain in performance for the joint model compared to the Cox model (Fig. 8). Therefore, in this example, there is no additional value for the more complex joint model.

## Discussion and outlook

Identifying patients at risk for disease as early as possible is crucial. More and more statistical methodology is published to perform this task as accurately as possible. One example is a joint model which combines longitudinal and survival data into a single model. Furthermore, the availability of primary care data is continually expanding, providing richer datasets for analysis [32]. However, it was unclear how the quality characteristics of real-life EHR data influences the performance of the joint models. In this study, we identified several critical parameters that relevantly influence the performance of the joint model. The joint model outperforms the conventional Cox regression model in scenarios where data contain noise and when the frequency of longitudinal measurements is increased. The homogeneity of the patients’ progress significantly influences the performance of the joint model, affected by both the count of patients showing an observable response and the consistency of the slope in the pre-disease phase. An increase in sample size substantially reduces the width of the prediction intervals derived from the iterated results in all models and, therefore, increases the accuracy. In total, there are certainly scenarios in which the joint model outperforms the Cox model - but also many scenarios in which no performance gain is observed. Therefore, the application of the more complex joint model, which also uses the full longitudinal trajectories, i.e., more information, needs to be considered carefully. For this purpose, we provide a checklist to allow the analyst to consider if the characteristics of the data offer the potential for a worthwhile application of the joint model.

When using real-world data to assess the predictive power of (longitudinal) measurements (bilirubin on PBC, eGFR on CKD) using the derived data quality guidelines, the expected outcome following the guidelines matches the performance outcomes. As all aspects are fulfilled for the first example, the joint model outperforms the Cox model in terms of the time-varying C-index, whereas for the second example not all requirements are fulfilled, causing that the joint model is not superior over the Cox model.

There are several further directions for research: the way in which the parameters interact with each other and how they may influence each other has not yet been analysed. For example, there is the possibility of a complex interplay between factors such as noise, sample size, and the effect being measured. Due to complexity considerations, an increased frequency of measurements over time, as is sometimes the case for specific markers, is not modelled. Additionally, due to computational time constraints, only a limited number of variations per parameter were considered in the analysis. This restriction suggests that a more thorough examination of a single parameter could benefit from simulating a larger number of values. Expanding the range of values for each parameter could lead to a more nuanced understanding of their effects and interactions, ultimately enhancing the robustness and reliability of the findings. The joint model can be further modified, as a linear slope after a specific break point is rarely observed in real-world data. Looking at complex real-world data, joint models can quickly become complex, especially when multiple events and multivariate longitudinal observations are involved [33]. For example, the parametrisation of the current value is used [5], i.e., directly including the longitudinal fit as a covariate for the survival submodel. However, alternative associations could be explored, such as interaction effects or time-dependent slopes [33].

**Fig. 10:**
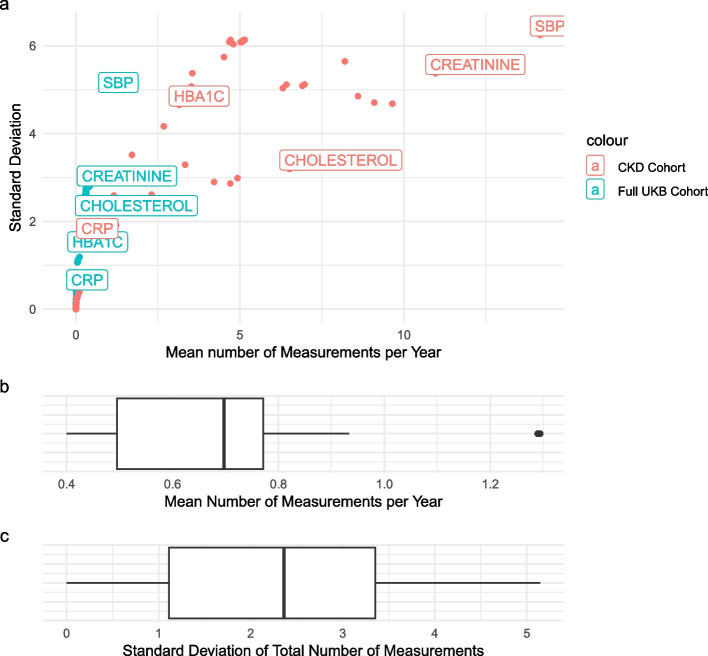
The mean number of measurements and the corresponding standard deviation for 66 preprocessed biomarkers in the UK Biobank Primary Care data are presented (**a**), with boxplots providing a summary for the mean (**b**) and the standard deviation (**c**). The analysis considers data collected from up to five years prior to the baseline visit through to the baseline visit itself. The data distinguished in red pertain exclusively to individuals with a CKD diagnosis, while the data in blue represent the entire cohort of UK Biobank participants with Primary Care Data

It may also be interested to gain a better understanding of the quality of EHR data between data sources and the health care system: so far, the data in the UK Biobank [34] have been used. There, data quality seems to be a challenge for the joint model. However, it is unclear how representative the EHR data is compared to other biobanks such as BioVU [35] and Singapore Precise [36]. Extracting data characteristics as shown here for a wider selection of EHR sources - and adjusting the parameters of the simulation study accordingly - would be a beneficial piece of research for making the checklist developed here even more broadly applicable. Given that different EHR systems can exhibit various challenges, including not only semantic discrepancies, but also issues related to data collection practices, it is essential to consider these factors when working with the data. To address system-specific impacts, a strong emphasis should be placed on preprocessing of the data. This may involve implementing additional outlier removal procedures and general quality control measures. If the established quality control parameters (see Appendix 1, Derivation of parameter choices based on real-world data section) remain insufficient, it may be necessary to explore further data smoothing techniques to improve data quality. Imputation methods have not been further investigated in this study. For the cox model, we use the last available measurement (i.e., no missingness expected) and the joint model is able to handle incomplete data. It should be noted that missingness is implicitly accounted for in the number of measurements included in the analysis in the joint model (see mean number of measurements in UK Biobank in Appendix 1, Fig. 10), i.e. the performance decreases if more observations are missing. Generally, it is important to note that the imputation of longitudinal data is a very complex topic and its implication on the conclusion presented in this paper may be a topic of future research.

As mentioned above, the presence of homogeneous subgroups is crucial to achieve optimal model performance. It is advantageous to specialise in more homogeneous subgroups to facilitate the detection of clearer signals. This can be accomplished not only through a knowledge-based approach that concentrates on a specific disease subgroup, but also by employing data-driven preprocessing techniques aiming to extract various clusters of longitudinal patterns [37]. To derive more meaningful and homogeneous clusters, one can utilise R packages such as *kml* and *kml3 d*, which offer implementations of k-means clustering specifically tailored for trajectory data (*kml*) or for joint trajectories (*kml3 d*) [38].

Furthermore, the application of this approach could extend to other areas of biostatistics, such as clinical trials that lead to a more consistent data collection and reduced variability, so that the trajectories are likely to be more homogeneous.

Ultimately, it may be interesting to establish similar guidelines for EHR data analysis for other complex modelling approaches.

This work and specifically the guidelines provided are valuable tools for identifying quality issues in EHR data and addressing the question of what data quality is required for the joint model. In addition, it can help in practical applications by providing insights on how to improve data quality, thereby improving the reliability and validity of joint modelling outcomes.

## Conclusions

The research indicates that joint models, which combine longitudinal and survival data, often surpass traditional Cox regression models, especially in situations with high levels of data noise and frequent longitudinal measurements. The efficacy of these joint models is highly dependent on the uniformity of patient progress, as indicated by consistent observable responses among patients. Moreover, larger sample sizes contribute to narrower prediction interval widths, improving accuracy across all models. Although joint models may excel in certain scenarios, there are cases where they do not outperform Cox models, requiring careful consideration before use. Furthermore, the research formulated guidelines for evaluating the quality of real-world data, demonstrating that following these guidelines can accurately forecast joint model performance.

The study offers valuable guidelines to ensure data quality, helping researchers and clinicians select suitable models. By evaluating joint models alongside traditional methods, the research enhances statistical methodologies in clinical studies, guiding analysts in choosing the right techniques based on data set characteristics. Ultimately, this study increases the reliability of healthcare data analysis, contributing to improved health outcomes.

## Figures and Tables

**Fig. 9:**
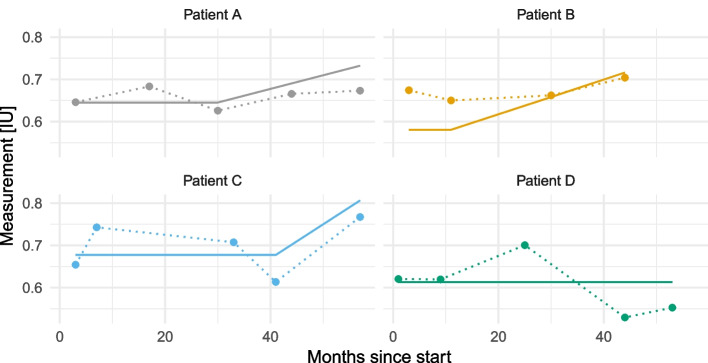
Example of four trajectories (solid line) assuming a piecewise linear structure with individual break points at $b_A=30,b_B=11, b_C=44, b_D>60$. Dashed line/dots mark hypothetical measurements

## Data Availability

The simulated datasets used and/or analysed during the current study are available from the corresponding author on reasonable request. The real-world data used can be accessed via the R-package JMbayes2 and by the UK Biobank. The UK Biobank data are not publicly available. Access to the UK Biobank data is available to researchers who apply for permission through the UK Biobank’s application process. For more information on accessing the data, please visit the UK Biobank website at https://www.ukbiobank.ac.uk. The program codes and data sets for the simulation study and illustrations are available on Zenodo with the ID 15296132 (DOI: 10.5281/zenodo.1529613210.5281/zenodo.15296132).

## Appendix 1

### Additional details on methodology

#### Simulation of the baseline characteristics

Baseline covariates are generated based on existing literature to mimic real-world scenarios. Specifically, the covariates included in the simulation were sex, age, and smoking status. These standard factors were chosen due to their common causalities with a broad range of diagnoses. For example, it is well established that smoking increases the risk of certain diseases, such as lung cancer [39]. By incorporating these covariates into the simulation, the study aims to better reflect the complexity of real patient data and to assess how these factors influence the onset and progression of the disease.

The distribution assumptions for the covariates given in Table 5 were used to evaluate the different influences of the baseline characteristics on the status of the disease.

**Table 5:** Distribution assumptions for simulated covariates; for sex: 0=female, 1=male; for smoking status: 1=currently, 0=never (patients who stopped smoking ignored)

Covariable	Distribution in healthy patients	Distribution in sick patients
Age	$58+\mathcal {N}(2,6)$	$62+\mathcal {N}(2,6)$
Sex	$Bin\left( 1/3\right)$	$Bin\left( 2/3\right)$
Smoking Status	$Bin\left( 2/5\right)$	$Bin\left( 2/3\right)$

#### UK Biobank

The UK Biobank consists of approximately 500,000 community-based participants aged 40 to 69 years, who were recruited throughout the UK between 2006 and 2010. All participants provided their informed written consent to participate in the study, which was approved by the National Research Ethics Service (11/NW/0382). Our study was conducted under access approval number 28807. In this study a variety of data were collected, including hospital inpatient data for the entire cohort and primary care records (including prescribed medications and coded clinical events, including consultations, diagnoses, procedures, and laboratory tests) for approximately 230.000 participants [34].

#### Derivation of parameter choices based on real-world data

Given individual measurements $y(t_{ij})$ with their respective time points $t_{ij}$ and diagnosis time point $t_{i,abs}$, the break point $t_i^{(b)}$ is defined as the specific time at which a significant change occurs in the behaviour of the measurements $y(t_{ij})$ for individual *i*. For now, it is assumed that the trend after reaching the break point is linear, as illustrated in Fig. 9. Given a data set, we can estimate the parameters mentioned in the chapter Framework for Simulating Longitudinal Primary Care Data by fitting a piecewise linear mixed-effects model based on the sick cohort. Therefore, we define $x_{1,i}(t)$ as the piece before the slope starts, resp. before the measurements are influenced by future diagnosis, and $x_{2,i}(t)$ as a piece when the slope is starting, resp. the measurements are influenced by future diagnosis,

$$\begin{aligned} x_{1,i}(t)= \left\{ \begin{array}{ll} t & ,t<b_i\\ 0 & , t\ge b_i \end{array}\right. ,\ \ \ \ x_{2,i}(t)= \left\{ \begin{array}{ll} t-b_i & ,t\ge b_i\\ 0 & , t< b_i \end{array}\right. \end{aligned}$$

.

The piecewise linear mixed-effects model is then defined as

$$\begin{aligned} y_i(t) & =x_{1,i}(t)+\tilde{x}_{2,i}(t)+\epsilon _i(t)\\ \tilde{x}_{2,i}(t) & =x_{2,i}(t)+\eta _i \ \ \ . \end{aligned}$$

An individual effect term, denoted as $\eta _i$, is incorporated to capture the individual slope term in the trajectory following the break point. The estimates given real-world data can be utilised for subsequent simulations, e.g. to analyse the data quality and its influence on further predictions. The analysis is carried out using the R package lme4 [40].

#### Preprocessing of eGFR trajectories within UK Biobank GP data

To effectively utilise the biomarker data of the Electronic Health Record (EHR), the simple extraction of biomarker measurements using read codes proves to be insufficient, and therefore a preprocessing pipeline must be applied.

The eGFR is calculated based on several time-independent characteristics of the patients and time-dependent levels of creatinine. More concretely, for deriving the EGFR value of a patient, the formula

16 $$\begin{aligned} \text {eGFR} & = 141 \cdot \min \left( \frac{\text {Scr}}{\kappa }, 1\right) ^{\alpha } \cdot \max \left( \frac{\text {Scr}}{\kappa }, 1\right) ^{-1.209} \cdot 0.993^{\text {Age}} \cdot \text {[1.018 if female]}\nonumber \\ & \quad \cdot \text {[1.159 if Black]} \end{aligned}$$

given by [41] is used. This equation calculates the estimated glomerular filtration rate (eGFR) using the CKD-EPI formula, where Scr is the serum creatinine concentration, $\kappa$ is 0.7 for women and 0.9 for men, and $\alpha$ is -0.329 for women and -0.411 for men. The serum creatinine concentration within the UK Biobank is given by five different readcodes, containing two different units (umol/L and mmol/L). Units are aligned prior to the calculation of the eGFR. The read codes are given by clinical terms using version 3 (CTV3 or v3). We use the recommended creatinine range for outlier exclusion given via the UK Biobank Showcase (see https://biobank.ndph.ox.ac.uk/showcase/field.cgi?id=30700, accessed: 5.12.2024) by $\left[ 10.7, 127.3\right]$. In order to restrict to the most relevant range of eGFR values (and to allow for a min-max normalisation which is only reasonable after the removal of outliers [42]), we include measurements within the interval $\left[ 1, 150 \right]$.

For anonymisation purposes, if the combination of unit and read code appears in fewer than five subject identifiers (SIDs), the associated SIDs should be removed.

## Appendix 2

### Supplementary tables

**Table 6:** ICD9 and ICD10 codes utilized in the definition of chronic kidney disease patients

ICD9	585, 5859, 4030, 4031, 4039, 4040, 4041, 4049
ICD10	I120, I131, I132, N18, N180, N181, N182, N183, N184, N185, N188, N189

## References

[1] Rizopoulos D. Dynamic Predictions and Prospective Accuracy in Joint Models for Longitudinal and Time-to-Event Data. Biometrics. 2011;67(3):819–29. 10.1111/j.1541-0420.2010.01546.x. 21306352 10.1111/j.1541-0420.2010.01546.x

[2] Scherrer JF, Pace WD. Will electronic health record data become the standard resource for clinical research? Fam Pract. 2017;34(5):505–7. 10.1093/fampra/cmx055. 28633339 10.1093/fampra/cmx055

[3] Zhang D, Shen D, Initiative ADN. Predicting Future Clinical Changes of MCI Patients Using Longitudinal and Multimodal Biomarkers. PLOS ONE. 2012;7(3):1–15. 10.1371/journal.pone.0033182. 10.1371/journal.pone.0033182 PMC3310854 22457741

[4] Tsiatis AA, Davidian M. Joint modeling of longitudinal and time-to-event data: an overview. Stat Sin. 2004;14(3):809–34.

[5] Ibrahim JG, Chu H, Chen LM. Basic Concepts and Methods for Joint Models of Longitudinal and Survival Data. J Clin Oncol. 2010;3:2796–801. 10.1200/JCO.2009.25.0654 PMC4503792 20439643

[6] Meiman J, Freund JE. Large data sets in primary care research. Ann Fam Med. 2012;10(5):473–4. 10.1370/afm.1441. 22966114 10.1370/afm.1441 PMC3438219

[7] Botsis T, Hartvigsen G, Chen F, Weng C. Secondary Use of EHR: Data Quality Issues and Informatics Opportunities. Summit Transl Bioinforma. 2010;2010:1–5. PMC3041534 21347133

[8] Therneau TM, Grambsch PM, Therneau TM, Grambsch PM. The cox model. New York: Springer; 2000.

[9] Albert PS. A linear mixed model for predicting a binary event from longitudinal data under random effects misspecification. Stat Med. 2012;31(2):143–54. 22081439 10.1002/sim.4405 PMC3874234

[10] Zhang J, Kim S, Grewal J, Albert PS. Predicting large fetuses at birth: do multiple ultrasound examinations and longitudinal statistical modelling improve prediction? Paediatr Perinatal Epidemiol. 2012;26(3):199–207. 10.1111/j.1365-3016.2012.01261.x PMC3324111 22471679

[11] Pinheiro J, Bates D, R Core Team. nlme: Linear and Nonlinear Mixed Effects Models. 2022. R package version 3.1-157. https://CRAN.R-project.org/package=nlme. Accessed 2 Dec 2024.

[12] Therneau TM. A Package for Survival Analysis in R. 2022. R package version 3.3-1. https://CRAN.R-project.org/package=survival. Accessed 2 Dec 2024.

[13] Rizopoulos D. The *R* Package JMbayes for Fitting Joint Models for Longitudinal and Time-to-Event Data Using MCMC. J Stat Softw. 2016;72(7). 10.18637/jss.v072.i07.

[14] Rustand D, Van Niekerk J, Krainski ET, Rue H, Proust-Lima C. Fast and flexible inference for joint models of multivariate longitudinal and survival data using integrated nested Laplace approximations. Biostatistics. 2024;25(2):429–48. 37531620 10.1093/biostatistics/kxad019 PMC11017128

[15] Rizopoulos D, Papageorgiou G, Miranda Afonso P. JMbayes2: Extended Joint Models for Longitudinal and Time-to-Event Data. 2024. https://github.com/drizopoulos/JMbayes2. Accessed 2 Dec 2024.

[16] Park SY, Park JE, Kim H, Park SH. Review of statistical methods for evaluating the performance of survival or other time-to-event prediction models (from conventional to deep learning approaches). Korean J Radiol. 2021;22(10):1697. 34269532 10.3348/kjr.2021.0223 PMC8484151

[17] Brentnall AR, Cuzick J. Use of the concordance index for predictors of censored survival data. Stat Methods Med Res. 2018;27(8):2359–73. 27920368 10.1177/0962280216680245 PMC6041741

[18] R Core Team. R: A Language and Environment for Statistical Computing. Vienna, Austria. 2022. https://www.R-project.org/. Accessed 2 Dec 2024.

[19] Wickham H, François R, Henry L, Müller K, Vaughan D. dplyr: A Grammar of Data Manipulation. 2023. R package version 1.1.4. https://CRAN.R-project.org/package=dplyr. Accessed 2 Dec 2024.

[20] Wickham H. ggplot2: Elegant Graphics for Data Analysis. Springer; 2016. https://ggplot2.tidyverse.org. Accessed 2 Dec 2024.

[21] Pedersen TL. patchwork: The Composer of Plots. 2024. R package version 1.2.0. https://CRAN.R-project.org/package=patchwork. Accessed 2 Dec 2024.

[22] Wickham H, Henry L. purrr: Functional Programming Tools. 2023. R package version 1.0.2. https://CRAN.R-project.org/package=purrr. Accessed 2 Dec 2024.

[23] Wickham H, Vaughan D, Girlich M. tidyr: Tidy Messy Data. 2024. R package version 1.3.1. https://CRAN.R-project.org/package=tidyr. Accessed 2 Dec 2024.

[24] Ho DE, Imai K, King G, Stuart EA. MatchIt: Nonparametric Preprocessing for Parametric Causal Inference. J Stat Softw. 2011;42(8):1–8. 10.18637/jss.v042.i08.

[25] Xie Y, Bowe B, Xian H, Balasubramanian S, Al-Aly Z. Renal function trajectories in patients with prior improved eGFR slopes and risk of death. PloS ONE. 2016;11(2):e0149283. 26900691 10.1371/journal.pone.0149283 PMC4762675

[26] Therneau TM, Grambsch PM. Modeling Survival Data: Extending the Cox Model. New York: Springer; 2000.

[27] Dickson ER, Grambsch PM, Fleming TR, Fisher LD, Langworthy A. Prognosis in primary biliary cirrhosis: model for decision making. Hepatology. 1989;10(1):1–7. 2737595 10.1002/hep.1840100102

[28] Markus BH, Dickson ER, Grambsch PM, Fleming TR, Mazzaferro V, Klintmalm GBG, et al. Efficacy of liver transplantation in patients with primary biliary cirrhosis. N Engl J Med. 1989;320(26):1709–13. 2659986 10.1056/NEJM198906293202602

[29] Tangri N, Inker LA, Hiebert B, Wong J, Naimark D, Kent D, et al. A Dynamic Predictive Model for Progression of CKD. Am J Kidney Dis. 2017;69(4):514–20. 10.1053/j.ajkd.2016.07.030. 27693260 10.1053/j.ajkd.2016.07.030

[30] Echouffo-Tcheugui JB, Kengne AP. Risk Models to Predict Chronic Kidney Disease and Its Progression: A Systematic Review. PLOS Med. 2012;9(11):1–18. 10.1371/journal.pmed.1001344. 10.1371/journal.pmed.1001344 PMC3502517 23185136

[31] Stroganov O, Fedarovich A, Wong E, Skovpen Y, Pakhomova E, Grishagin I, et al. Mapping of UK Biobank clinical codes: Challenges and possible solutions. PLoS ONE. 2022;17(12):1–15. 10.1371/journal.pone.0275816. 10.1371/journal.pone.0275816 PMC9757572 36525430

[32] Office of the National Coordinator for Health Information Technology. National trends in hospital and physician adoption of electronic health records. HealthIT. gov. 2022.

[33] Lawrence Gould A, Boye ME, Crowther MJ, Ibrahim JG, Quartey G, Micallef S, et al. Joint modeling of survival and longitudinal non-survival data: current methods and issues. Report of the DIA Bayesian joint modeling working group. Stat Med. 2015;34(14):2181–95. 10.1002/sim.6141. 24634327 10.1002/sim.6141 PMC4677775

[34] Sudlow C, Gallacher J, Allen N, Beral V, Burton P, Danesh J, et al. UK Biobank: An Open Access Resource for Identifying the Causes of a Wide Range of Complex Diseases of Middle and Old Age. PLOS Med. 2015;12(3):1–10. 10.1371/journal.pmed.1001779. 10.1371/journal.pmed.1001779 PMC4380465 25826379

[35] Center VUM. BioVU: The Vanderbilt University Medical Center Biobank. victr.vumc.org/biovu-description/. Accessed 19 Nov 2024.

[36] Singapore PHR. PRECISE. https://www.npm.sg/. Accessed 19 Nov 2024.

[37] Qiu J, Hu Y, Li L, Erzurumluoglu AM, Braenne I, Whitehurst C, et al. Deep representation learning for clustering longitudinal survival data from electronic health records. Nat Commun. 2025;16(1):2534. 40087274 10.1038/s41467-025-56625-z PMC11909183

[38] Genolini C, Alacoque X, Sentenac M, Arnaud C. kml and kml3d: R packages to cluster longitudinal data. J Stat Softw. 2015;65:1–34.

[39] Walser T, Cui X, Yanagawa J, Lee J, Heinrich E, Lee G, et al. Smoking and lung cancer: the role of inflammation. Proc Am Thorac Soc. 2008;5(8):811–5. 10.1513/pats.200809-100TH. 19017734 10.1513/pats.200809-100TH PMC4080902

[40] Bates D, Mächler M, Bolker B, Walker S. Fitting Linear Mixed-Effects Models Using lme4. J Stat Softw. 2015;67(1):1–48. 10.18637/jss.v067.i01.

[41] Levey AS, Stevens LA, Schmid CH, Zhang Y, Castro AF III, Feldman HI, et al. A new equation to estimate glomerular filtration rate. Ann Intern Med. 2009;150(9):604–12. 19414839 10.7326/0003-4819-150-9-200905050-00006 PMC2763564

[42] Vafaei N, Ribeiro RA, Camarinha-Matos LM. Comparison of Normalization Techniques on Data Sets With Outliers. Int J Decis Support Syst Technol (IJDSST). 2022;14(1):1–17.

